# Telepathy and Telegraphy

**Published:** 1910-10

**Authors:** 


### EDITORIAL DEPARTMENT.

### TELEPATHY AND TELEGRAPHY.

In the *Lancet-Clinic* for September there is an editorial on Telepathy which so impressed me that I am induced to say a few words on the subject, and to reproduce the paper which follows. I "went on record" on this subject some ten years ago, and I am pleased to see that the talented editor of the *L.-C.* "comes across" so gracefully and expresses views,—so far as they go,—in accordance with those long held by myself.

The paper herewith published was one of twenty contributed some eight or ten years ago to a "Symposium" gotten up by Hon. Clark Bell, of New York, and published by him in book form and also in the *Medico-Legal Journal,* of which he is the editor and publisher. It has never been published elsewhere, so far as I know. The "Symposium" was intended to show the views of psychologists—evoked by Mrs. Piper's disclaimer in the *New York Herald* of the allegations that she was "controlled by 'spirits.'" The contributors were Thompson J. Hudson, the late Prof. Wm. James, Professor Hyslop and others, the writer of this amongst them. The writer has also incorporated his views on the subject, in a recent publication in book form, "The Strange Case of Dr. Bruno."

I hold that telepathy and wireless telegraphy and telephony are identical, and that the mechanism of the latter is an unconscious duplicature of the nervous system of man, just as all of our mechanical devices are copied from God's masterpiece. The skull is the arch, the strongest feature in architecture; the muscles and bones are the levers and pulleys; the heart and veins are the pump and valves; the Holly system of waterworks and the Warring system of sewers are an exact duplicature of the circulatory system of man; the lungs is the bellows, and our entire electrical system is a duplicate af the nervous system, the brain being the original dynamo whereby a part of the general energy which is expressed in all the phases of animal life, is converted into electrical energy and is expressed in thought (and all mental processes), which, being projected into space (an electrical force—vibrations), reaches the person for whom the "message" (if in that form) is intended, if that person be *en rapport* with the sender, just as Mr. Marconi generates electricity artificially, and the wireless operator sends it into space, to be received by the machine keyed to corresponding wave lengths. It is quite common for close friends to *sym-pathize*—at a distance, and I make bold to express the belief that man of the future,—near future,—will be able to communicate by telepathy. Thought transference, clairvoyance and clairaudience are facts. The *Lancet-Clinic* writer has pressed the button and started the machinery here: but I must "ring off." Read the following:

